# Processing and Characterization of Nanoparticle Coatings for Quartz Crystal Microbalance Measurements

**DOI:** 10.6028/jres.120.001

**Published:** 2015-01-30

**Authors:** Jessica D. Torrey, Teresa L. Kirschling, Lauren F. Greenlee

**Affiliations:** 1 Technical Service Center of the US Bureau of Reclamation, Lakewood, CO, 80225; 2 National Institute of Standards and Technology, Boulder, CO, 80305; 3 United States Geological Survey, Lakewood, CO, 80225

**Keywords:** coatings, nanoparticles, quartz crystal microbalance, titanium dioxide, zero-valent iron

## Abstract

The quartz-crystal microbalance is a sensitive and versatile tool for measuring adsorption of a variety of compounds (e.g. small molecules, polymers, biomolecules, nanoparticles and cells) to surfaces. While the technique has traditionally been used for measuring adsorption to flat surfaces and thin ridged films, it can also be extended to study adsorption to nanoparticle surfaces when the nanoparticles are fixed to the crystal surface. The sensitivity and accuracy of the measurement depend on the users’ ability to reproducibly prepare a thin uniform nanoparticle coating. This study evaluated four coating techniques, including spin coating, spray coating, drop casting, and electrophoretic deposition, for two unique particle chemistries [nanoscale zero valent iron (nZVI) and titanium dioxide (TiO_2_)] to produce uniform and reproducible nanoparticle coatings for real-time quartz-crystal microbalance measurements. Uniform TiO_2_ coatings were produced from a 50 mg/mL methanol suspension via spin coating. Nanoscale zero-valent iron was best applied by spray coating a low concentration 1.0 mg/mL suspended in methanol. The application of multiple coatings, rather than an increase in the suspension concentration, was the best method to increase the mass of nanoparticles on the crystal surface while maintaining coating uniformity. An upper mass threshold was determined to be approximately 96 µg/cm^2^; above this mass, coatings no longer maintained their uniform rigid characteristic, and a low signal to noise ratio resulted in loss of measurable signal from crystal resonances above the fundamental.

## 1. Introduction

Measurements of adsorption and reaction at surfaces are particularly important in a variety of fields including catalysis, corrosion, polymer and surfactant science and biological engineering [1–3]. Many times, the surface of interest is a nanoparticle surface instead of a macroscopic flat surface, and the measurement needs to take place in a complex aqueous environment. Measuring adsorption and reaction at nanoparticle surfaces in real time is more difficult than performing the same measurements on thin films. Common techniques for adsorption measurements [surface plasmon resonance (SPR), ellipsometry, total internal reflection fluorescence (TIRF), and attenuated total reflection Fourier transform infrared spectroscopy (ATR-FTIR)] [4–7] are designed to be performed on flat surfaces. Solution depletion methods are often used to determine adsorbed mass on nanoparticle surfaces [8], however this time intensive method does not allow the user to easily examine the adsorption kinetics. The quartz crystal microbalance (QCM) is a versatile technique for sensitively detecting mass changes at surfaces [1–3, 9–12]. If nanoparticles can be uniformly deposited on the sensor surface, the QCM has the potential to measure mass changes on the surfaces of nanoparticles, which would provide valuable information for a range of fields.

Since the original paper by Sauerbrey in 1959, use of the QCM has proven to be a versatile technique for measuring small mass changes [9]. With mass sensitivities of commercial instruments on the order of nanograms per square centimeter, the QCM has been employed particularly in the biological and environmental fields to study real-time surface adhesion and deposition [2, 11]. While the microbalance technique is used extensively in monitoring adsorption to monolayers and thin films [13–16], few researchers have used QCM as a tool to monitor real-time changes at nanoparticle surfaces. Despite the lack of studies, there is significant evidence that QCM can provide valuable information about adsorption and reaction at nanoparticle surfaces. QCM has been shown to be sensitive to nanoparticle coatings, for example in the cases of measurement of nanoparticle concentrations and microscale thermogravimetric analysis [1, 12], and reactions on the surface of thin films have also been investigated by QCM [13, 16].

QCM relies on the piezoelectric properties of quartz; with the application of an electric field, mechanical strain is created in the crystal. In an AT-cut quartz plate, an oscillating voltage applied to electrodes on opposite surfaces will excite resonant through-thickness shear modes, if the frequency of the driving voltage matches that of one of these modes. Mass loading of the crystal surface will change the resonant frequencies. Sauerbrey [9] derived a relationship (Eq. (1)) between frequency change (Δ*f*) and mass change per unit area (Δ*m*) for a rigidly adsorbed uniform elastic thin film:

$$\Delta f=\frac{f\cdot \Delta m}{d\cdot \rho_{q}}=-C_{f}\cdot \Delta m,$$ (1)

where *f* is the resonance frequency of a through-thickness mode, *d* is the crystal thickness, and *ρ_q_* is the density of quartz. The mass sensitivity factor *C_f_* = 5.66 × 10^6^ Hz·m^2^/kg when *f* = 5 MHz. Because the Sauerbrey relationship was developed specifically for thin films that can be considered as an extension of the crystal, to perform measurements of adsorption onto nanoparticles, care must be taken to ensure that a thin (< 1 µm) uniform particulate coating is applied to the quartz crystal surface [10, 12].

One specific application where measurements at nanoparticle surfaces in complex environments are needed is in the water treatment field. Nanotechnology is increasingly being proposed for a variety of water treatment applications [17–21]. Two of the most common nanoparticle types studied are titanium dioxide (TiO_2_) photocatalytic nanoparticles and nanoscale zero-valent iron (nZVI) reactive particles. These materials react in aqueous environments with contaminants ranging from endocrine disrupting compounds to disinfection by-products to heavy metals [18, 22, 23]. For both systems, the adsorption of contaminants to the nanoparticle’s surface plays a key role in the contaminant degradation process. Additionally for nZVI, the kinetics of the iron oxidation reaction in water determines the useful lifetime of this material as a decontaminant [24]. To fully understand the adsorption and oxidation behavior, we need to be able to quantify small mass changes occurring at the surface of the nanoparticle.

Many different coating techniques are used to deposit particles with a range of surface and particle chemistries onto QCM crystals, which can lead to differences in the quality of QCM measurements. The sheer number of potential combinations leads to several issues, including data reproducibility, and an inability to compare results obtained from different experimental groups. Therefore, in this work, we focus on the development of a robust and reproducible coating technique for QCM crystals. In this study, four coating methods were tested with two different nanoparticle systems, TiO_2_ and nZVI, to determine the most suitable methods for application of nanoparticles to a quartz crystal. The quality of the resulting coatings is highly dependent on the technique used, as well as the concentration of the starting suspension and the coatings parameters.

## 2. Experimental Procedure

### 2.1 Materials

Commercial TiO_2_ nanoparticles and nZVI particles that were synthesized in-house were used in these experiments. The TiO_2_ powder (Evonik AEROXIDE® P25)^1^ had an as-received mean particle diameter of 21 nm and a specific surface area of 50 m^2^/g. nZVI was synthesized according to previously published techniques [25, 26]. The particles had a monomodal mean particle diameter of approximately 100 nm [26] and an expected specific surface area of 18 m^2^/g to 21 m^2^/g [27, 28].

### 2.2 Suspension Preparation and Characterization

TiO_2_ powder was suspended in either ultrapure water at pH = 4 or methanol, in 20 mL batches. Suspension vials were immersed in an ice bath and sonicated with a 3 mm diameter probe for 15 min at 1.6 W and 80 % pulse. Following sonication, suspensions were measured using dynamic light scattering (DLS) and laser Doppler electrophoresis (LDE). In water at pH = 4, the TiO_2_ was determined by DLS to have an average agglomerate diameter (DLS data reported as z-average diameter) of 219 nm ± 2.25 nm and by LDE to have a zeta potential of 32.8 mV ± 1.28 mV. In methanol, the TiO_2_ had an average agglomerate diameter of 232 nm ± 4.03 nm and a zeta potential of 30.5 mV ± 0.91 mV. Three concentrations of TiO_2_ were tested in the subsequent procedures: 50 mg/mL, 10 mg/mL, and 1.0 mg/mL.

nZVI was precipitated in an aqueous solution from iron sulfate and sodium borohydride in 5 mL batches with carboxymethylcellulose (CMC) (250,000 g/mole, degree of substitution 0.7) as a stabilizer. To reduce oxidation, the suspensions were then centrifuged and the iron nanoparticles were resuspended in methanol. Suspensions were sonicated in a bath sonicator for 60 min at 95 W. Following sonication, suspensions were measured by use of DLS and LDE. In methanol, the nZVI was found to have an average agglomerate diameter of 184 nm ± 6 nm and a zeta-potential of −20.6 mV ± 2.9 mV. As with TiO_2_, three concentrations of nZVI were tested in subsequent procedures: 50 mg/mL, 10 mg/mL, and 1.0 mg/mL.

### 2.3 Coating Techniques

Four common coating techniques were evaluated in this study: drop casting, spin coating, spray coating, and electrophoretic deposition. When drop casting TiO_2_ suspensions, crystals were heated on a hot plate to the boiling point of the solvent: 100 °C for water and 65 °C for methanol. Crystals were not heated for nZVI suspensions, to avoid nanoparticle oxidation, which is accelerated at high temperatures. 10 µL of suspension was dropped onto the center of a crystal and allowed to dry. Crystals were cooled to room temperature before QCM measurement.

For spin coating, 25 µL of suspension was dropped onto a crystal spinning at 1000 rpm, 3000 rpm, or 6000 rpm. The samples were spun for one minute at this speed and then reduced to 600 rpm for one minute to allow complete evaporation of the solvent. Spray coated samples were prepared by use of a gravity-feed airbrush at 207 kPa (30 psi) nitrogen pressure. The outer edge of each crystal was masked so that only a centered circle of 10 mm diameter was coated. Crystals were sprayed for approximately 2 s from 15 mm distance. For both of these techniques, multiple coatings were applied to vary the mass of material on the crystal.

Finally, electrophoretic deposition was used to coat the crystals by use of an apparatus designed and built in-house. The two electrodes were held in place at a distance of 20 mm by gold plated clamps. These were lowered and raised at a constant speed into a borosilicate beaker containing 100 mL of the coating suspension. Before each coating process, the suspension was magnetically stirred or shaken, for TiO_2_ or nZVI, respectively. The electrodes were connected to a DC power source and a multimeter. When coating TiO_2_, which has a positive charge in water and methanol, the crystal substrate was placed at the cathode. When coating nZVI, which has a negative charge in methanol, the crystal substrate was placed at the anode. A Pt-coated quartz crystal was the counter electrode. To determine optimal coating conditions, both the applied voltage and time were varied. Samples were prepared at 1 V, 10 V, or 20 V for 15 s or 30 s. The highest concentration suspension, 50 mg/mL, resulted in a thick coating even at the lowest voltage and shortest time, so only 1.0 mg/mL and 10 mg/mL concentrations were tested using all parameters. After deposition, the crystals were rinsed with ultrapure water or methanol (matching the solvent used in deposition) to remove loose powder, and then air-dried.

### 2.4 Characterization of Coatings

The crystals were measured before and after coating in air by use of a QCM with dissipation monitoring (Q-Sense E4, Biolin Scientific AB, Sweden) to determine the amount of material deposited onto the crystal surface. Because the coatings were rigid and dissipation was relatively low (<1 × 10^−6^ per 10 Hz frequency shift), Sauerbrey modeling could be used to determine the mass loading on the crystal surface [9, 10]. AT-cut quartz crystals (Q-Sense QSX301 and QSX314, Biolin Scientific AB, Sweden) with a fundamental resonance frequency (*f_0_*) of 5 MHz were used for all experiments, and odd overtones (*n* = 3, 5, 7, 9, 11, and 13) were measured, in addition to *f_0_*, at T = 23 °C with averaging over 3 seconds. Gold electrodes were used for the experiments with nZVI; platinum electrodes were used for experiments with TiO_2_. Prior to coating with nZVI or TiO_2_, the crystals were cleaned by the following procedure: sonicate for 20 min in a mixture of acetone, methanol, and isopropanol, sonicate for 20 min in a mixture of ethanol and water, and UV-ozone (254 nm) clean for 20 min. Scanning electron microscopy (SEM) was performed on all the crystals to determine the morphology and uniformity of the applied coatings. Coatings were evaluated for particle dispersion, coating thickness, homogeneity of the surface coverage, and, finally, whether or not a coating produced a QCM signal up to the thirteenth harmonic.

## 3. Results

### 3.1 Morphology of TiO_2_ Coatings

**Fig. 1:**
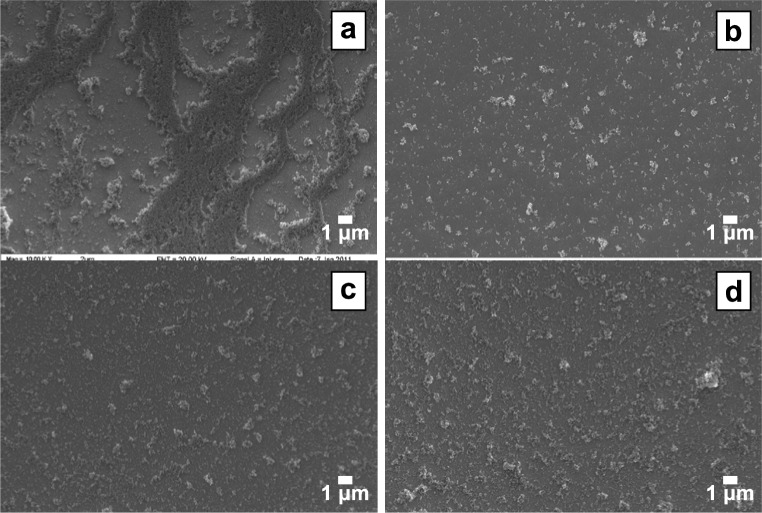
SEM micrographs of TiO_2_ nanoparticle coatings on a Pt-coated quartz crystal deposited from 10 mg/mL methanol suspension via (a) drop casting, (b) spin coating (3000 rpm), (c) spray coating, or (d) electrophoretic deposition (10 V, 30 s). The most effective technique for consistently depositing a uniform TiO_2_ coating was spin coating.

Drop casting was the simplest of the four techniques that were tested, but the quality and reproducibility of drop cast coatings is heavily dependent on operator capability. Care must be taken to drop the suspension from a consistent height as close to the center of the crystal as possible to produce samples with uniform coating. With both water and methanol solvents, the droplet diameter was approximately 4 mm. Droplets prepared with water as the solvent lead to a relatively uniform coating. Droplets prepared with methanol dried quickly in non-concentric rings, and this pattern resulted in an inhomogeneous surface coverage of the crystal with the bulk of the nanoparticles located in the outer edge of the coated circle (Fig. 1a). This pattern results from the “coffee ring” effect: contact line pinning and a capillary flow that drives the dispersed material to the edge of the droplet [29]. Varying the suspension concentration or lowering the casting temperature of the crystal did not significantly change these results. For coatings prepared with 10 mg/mL and 50 mg/mL water suspensions, drying cracks were observed, indicative of thick coatings; approximately 125 µg and 450 µg of TiO_2_, respectively, were deposited, based on the change in crystal mass. The most homogeneous coatings were those cast from 1.0 mg/mL concentration of TiO_2_ in water. These coatings had consistently macroscopically uniform distributions of the TiO_2_ particles across the droplet diameter, and no drying cracks were observed. The deposited mass ranged from approximately 15 µg to 20 µg.

The uniformity of TiO_2_ coatings prepared by spray coating was also poor at low suspension concentrations and with water as the medium. Coatings from 1.0 mg/mL or 10 mg/mL suspensions yielded inhomogeneous surface coverage when viewed on the macroscale (Fig. 1c). However, at 50 mg/mL and an intermediate concentration of 25 mg/mL, uniform particulate coatings could be easily reproduced. Deposited mass was determined, by QCM techniques and the Sauerbrey relationship, to be 30 µg and 145 µg per layer for 25 mg/mL and 50 mg/mL concentrations, respectively. The quality of spray coated films depends on a variety of parameters, including droplet size, evaporation rate, the contact angle of drops on the surface, and coalescence rate [30]. As with drop casting, this technique is dependent on the skill of the user, and some practice is required with the air gun before optimal results can be achieved.

Electrophoretic deposition was an efficient method for coating TiO_2_ on QCM crystals. Suspension solvent and concentration both played a strong role in affecting the coating morphology; the quality of coatings was more dependent on deposition voltage than time. In methanol, only samples prepared with 1.0 mg/mL suspension and 10 V bias created a uniform coating ranging from 8 µg to 14 µg TiO_2_ (Fig. 1d). In water at low concentrations of TiO_2_, uniform coatings were prepared that did not vary with deposition period and showed only a slight dependence on voltage from 1 V to 20 V. The best deposition parameters were water as the suspension medium at 10 V bias and either 15 s or 30 s deposition period. These coatings were macroscopically and microscopically uniform, with approximately 50 µg of TiO_2_ coated onto the crystal surface.

**Fig. 2:**
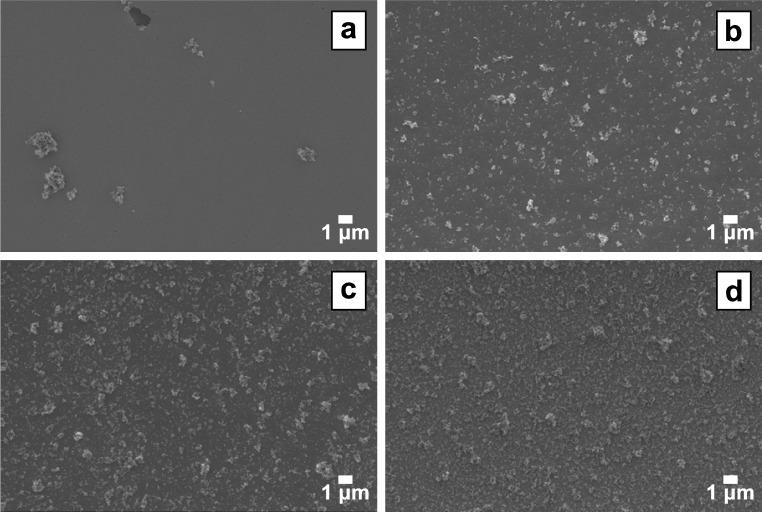
SEM images of TiO_2_ nanoparticles spin coated at four different concentrations at 3000 rpm: (a) 1.0 mg/mL, (b) 10 mg/mL, (c) 25 mg/mL, and (d) 50 mg/mL. The coatings made from the 50 mg/mL suspension were consistently uniform, with approximately 14 µg of TiO_2_ deposited per 25 µL of suspension.

Spin coating was the most effective method for depositing uniform TiO_2_ coatings onto QCM crystals (Fig. 1b). Initial attempts with water as a solvent resulted in poor coatings, because of the low vapor pressure of water at room temperature, so subsequent investigations were performed using only methanol suspensions. A spin speed of 1000 rpm did not yield high uniformity. For the lowest concentration tested, 1.0 mg/mL, little TiO_2_ remained on the crystal surface after spinning at any of the three speeds that were tested. However, at 10 mg/mL and especially 50 mg/mL, homogeneous particulate coatings were produced with good surface coverage at spin speeds of 3000 rpm and 6000 rpm (Fig. 2). A fourth concentration, 25 mg/mL, was also tested. For 50 mg/mL concentration and a spin speed of 3000 rpm, approximately 14 µg of TiO_2_ was deposited on the crystal per 25 µL drop of suspension.

### 3.2 Morphology of nZVI Coatings

**Fig. 3:**
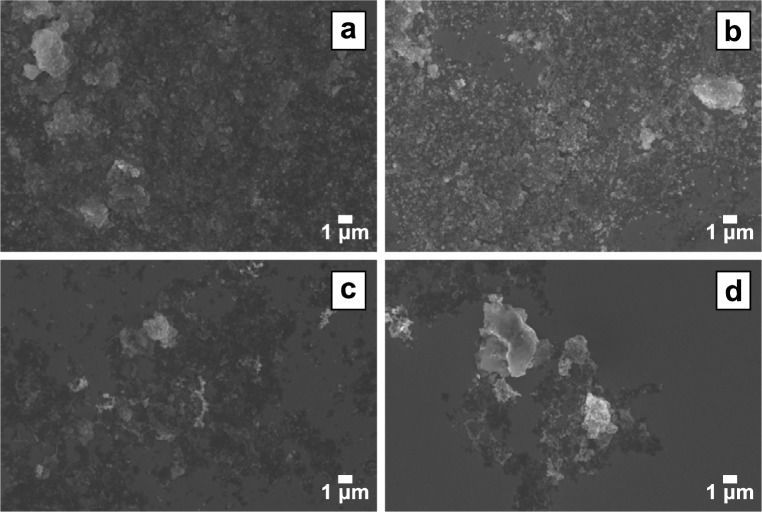
Comparison of SEM images of coatings produced with a 10 mg/mL nZVI particle suspension in methanol via (a) drop casting, (b) spin coating (3000 rpm), (c) spray coating, and (d) electrophoretic deposition (10 V, 30 s). The most effective technique for consistently depositing a uniform nZVI coating was spray coating.

Similar to TiO_2_ coating techniques described in the previous section, drop casting (Fig. 3a) was the easiest technique to use with nZVI samples. However, water is not a recommended solvent for depositing nZVI because the nanoparticles oxidize as the water evaporates from the sample. When nZVI nanoparticles are drop cast from a methanol suspension, the particles tend to dry and deposit on the surface in irregular patterns, again displaying the “coffee ring” effect. This coating method unevenly distributes particles on the surface of the crystal and agglomeration of the particles in a multilayered structure is evident in SEM images. An increase in the particle concentration further increased particle agglomeration and irregular particle deposition, as well as the total mass deposited, on the crystal surface. As a result, inconsistent results were obtained in subsequent QCM experiments. At a nanoparticle concentration of 1.0 mg/mL, approximately 6 µg of nanoparticles were deposited per layer. At the two higher nanoparticle concentrations (10 mg/mL and 50 mg/mL), the mass deposited on the crystal was greater than 100 µg. Therefore, if drop casting is chosen as a coating technique based on ease of use, the nanoparticle suspension should be prepared at a relatively low concentration (*e.g.*, 1.0 mg/mL).

Spin coating of nZVI particle suspensions in methanol resulted in less deposited mass than drop casting at similar nanoparticle concentrations. However, the distribution of nanoparticles on the surface of the crystal was quite similar; bare areas and areas of agglomerated nanoparticles were observed (Fig. 3b). An increase in the spin speed did not improve the distribution of nanoparticle deposition on the crystal surface, but it did result in a decrease in the deposited mass of nanoparticles. An increase in the nanoparticle concentration resulted in an increase in deposited mass, but none of the conditions tested resulted in a deposited mass of greater than 10 µg. While spin coating is the most reliable method for coating stable suspensions of TiO_2_ nanoparticles onto crystals, nZVI suspensions are far less stable which may result in the poor coverage. A suspension of nZVI (which is magnetic) can aggregate to micron sized aggregates in a matter of minutes [31].

Electrophoretic deposition of methanol suspensions of nZVI particles resulted in limited particle deposition on the crystal surface (Fig. 3d). While the zeta potential of the stabilized nZVI particles (−20.6 mV ± 2.9 mV) indicated that the particles were sufficiently charged and a candidate for electrophoretic deposition, the technique was unsuccessful at producing a well-distributed layer of particles. Increases in nanoparticle concentration, deposition period, or deposition voltage increased particle deposition no further. This result may be caused by preferential deposition of free CMC, which is also charged and has a larger diffusion coefficient than the particles.

**Fig. 4:**
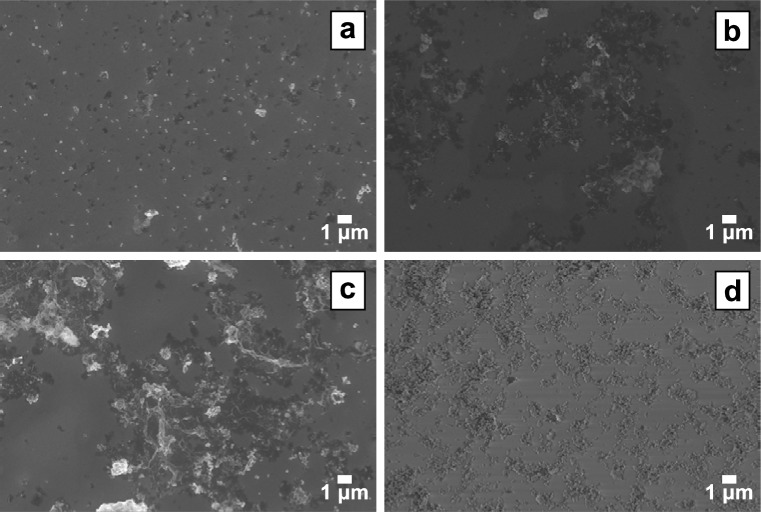
SEM images of stabilized nZVI particles spray coated at three different nanoparticle concentrations, (a) 1.0 mg/mL, (b) 10 mg/mL, and (c) 50 mg/mL. The lowest nanoparticle concentration, 1.0 mg/mL, was then used in (d) to obtain single layer coatings of the iron nanoparticles through repeated sprays (seven coats shown).

In contrast to drop casting, spin coating, and electrophoretic deposition, the spray coating method, used with methanol suspensions, resulted in an evenly distributed coating of nZVI particles. However, spray coating at higher concentrations did not improve nanoparticle distribution or the amount of nanoparticles on the crystal surface (Fig. 3c, organic stabilizer may appear as the darker areas on crystal surface). A comparison of the three nanoparticle concentrations (1.0 mg/mL, 10 mg/mL, and 50 mg/mL) can be seen in Fig. 4a–c. After this comparison showed that the lowest nanoparticle concentration resulted in well-distributed nanoparticle deposition, multiple rounds of spray coating at 1.0 mg/mL were used to obtain increased surface coverage (Fig. 4d). Multiple coatings did not result in complete surface coverage but did achieve a single-particle-thick coating. The technique of spray coating, with a low nanoparticle concentration in methanol and the application of several coats, was determined to be the optimal method for deposition of nZVI particles on QCM gold-coated crystals. Lower particle concentrations aggregate slower than higher concentrations, and it is possible that the high shear environment in the air gun disrupts aggregates during the coating process leading to more uniform coatings.

### 3.3 QCM Characterization of Coatings

**Fig. 5:**
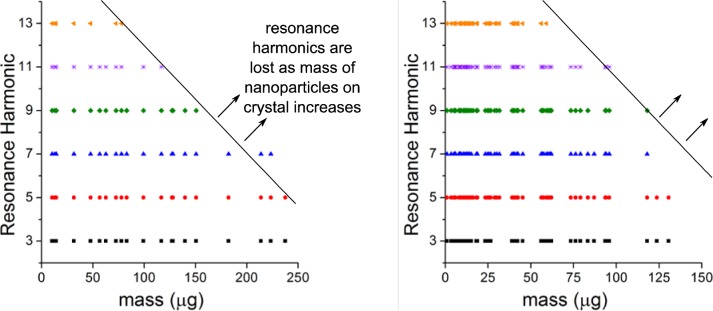
Disappearance of resonance harmonics due to an increase in nanoparticle mass loading on QCM crystals. Both types of nanoparticles (left graph is TiO_2_, right graph is nZVI) resulted in similar behavior as nanoparticle mass increased on the crystal.

In addition to analyzing the morphology of the nanoparticle coatings, each set of coating parameters was also tested in the QCM. A completely homogeneous, well-adhered, rigid thin film will lead to a high signal-to-noise ratio and measurable resonances up to at least the thirteenth harmonic with less than ±3 Hz deviation in frequency. Nonuniformity in the coating can cause increased damping (and associated loss of signal) through coupling to spurious modes of the crystal. Additionally, large mass loading can also lead to damping and result in a loss of signal at the higher harmonics (there is not a high enough power input to drive the crystal to high frequencies). Therefore, the number and quality of measurable resonances for a particular set of coating parameters can be used as a metric for coating quality. For example, although TiO_2_ coatings produced by spin coating and spray coating appeared to have similar morphology in the SEM analysis, loss of signal from the eleventh and thirteenth resonance harmonics for the spray coated crystals indicated that these coatings were not uniform over the entire area of the crystal. Coating parameters were optimized for both nanoparticle systems, and these parameters were then used to coat multiple layers of nanoparticles and identify the mass threshold above which resonance signals were lost. TiO_2_ was coated via spin coating at 3000 rpm from a 50 mg/mL methanol suspension, yielding approximately 14 µg per layer. nZVI was coated via spray coating from a 1.0 mg/mL methanol suspension, yielding 5 µg to 10 µg per layer. For both TiO_2_ and nZVI nanoparticles, the upper limit for sample mass was approximately 75 µg (Fig. 5), or 96 µg/cm^2^. Above this mass, at least one of the resonant harmonics was not measurable. Typically, the harmonics were lost in descending order, with the thirteenth harmonic lost first. Based on these results with two different nanomaterials, this upper mass limit can be used as a general guideline to prepare nanoparticle-based samples for QCM analysis.

## 4. Conclusions

This study has evaluated four coating techniques and several processing parameters in order to optimize nanoparticle coatings on gold- and platinum-coated quartz crystals. The goal of coating optimization was to obtain accurate and repeatable results when a quartz crystal microbalance is used as a tool to measure real-time adsorption and reaction at nanoparticle surfaces. Titanium dioxide and zero-valent iron nanoparticles were coated on QCM crystals through drop casting, spin coating, spray coating, and electrophoretic deposition. For the TiO_2_ nanoparticles, uniform coatings were produced from 50 mg/mL methanol suspension by spin coating at 3000 rpm. With nZVI nanoparticles, the best coatings were achieved by spray coating a low concentration 1.0 mg/mL methanol suspension. The application of multiple coatings, rather than an increase in the suspension concentration, was the best method to increase the mass loading on the crystal. The upper mass threshold for QCM measurements for both TiO_2_ and nZVI coated crystals was approximately 96 µg/cm^2^.

## Footnotes

1 Commercial equipment, instruments, or materials are identified only in order to adequately specify certain procedures. In no case does such identification imply recommendation or endorsement by the National Institute of Standards and Technology, nor does it imply that the products identified are necessarily the best available for the purpose.
